# Quackery and Its Cure

**Published:** 1859-10

**Authors:** A. G. Thomas

**Affiliations:** Atlanta, Georgia


### ARTICLE IV.

*Quackery and its Cure!—An Essay read before the Medical Association of the State of Georgia, April,* 1859. By A. G. Thomas, A. M., M. D., of Atlanta, Georgia. Printed by order of the Association.

The history of intellect is summed up in the one word, PROGRESS. In every department of science the march of mind has been onward and upward; hints have been seized by the investigating genius, and by the careful consideration of small things vast structures of science have been erected to perpetuate the active industry and unbending energy of those who were bold enough to think, and thinking to experiment, and experimenting to develop truths and declare them to the world. To-day science stands upon a firmer basis than it ever has before, and universal activity of intellect is the characterizing spirit of the present age. But while the battle-axe of Truth is being nobly and successfully wielded against established errors, over-wrought enthusiasm pushes its inquiries beyond the proper limits of investigation, and results in a maze of theoretical mysticisms. Speculation, with chameleon face, starts up in every lane of learning, and with eager boldness, confronts the fixed principles of science. Systems of pseudo-philosophy, a fearful number treading fast on the heels of each other, overleap the confines of common sense, annul the laws of reason, and unblushingly attack the strong-holds of Truth; yet, though it be true, that theorizers flood the world with the frothy foment of a chaotic philosophy; the sciences are advocated by a host of noble spirits, and stand forth bright and unsullied amid all this fog of foolery; and none stands higher in the catalogue than that of Medicine, though it be not altogether disencumbered of the direful incubus of hydra-headed Error. While we believe it to be true, that Medicine has collected such a mass of facts, and established such a number of principles, as to rank her foremost among the sciences, yet we are compelled to see that a very low estimate is put upon the medical profession, by the many of the educated and hosts of the illiterate. Why is it that this unfounded and unreasonable estimate has been made of Medicine? To this question we offer the answer, that such a thing exists as *Quackery.* It is to the consideration of a few of the phases of this hundred-headed, thousand-handed, color-changing *thing*, that I invite the members of this society.

*Quackery* may be defined exclusiveism or one ideaism, and the chief idea in most of the specimens, is to make money. The first view which I shall take, is of what may be termed Humbug Quackery. This bug I shall not attempt to dissect anatomically and present the origin, insertion and relation of its various parts, for I am satisfied, that all present have seen his *tracks* often enough to recognize the animal, if we only show up a section of one or two regions of its gigantic structure. Of those lying parasites of medicine, which their scoundrelly authors have showered over the land, the thousand and one cure-everything-under-the-sun, oils, ointments and opiates; potions, plasters, pills and powders, until they are as numerous as bed-bugs in a country village hotel, it is only necessary to say, that they are so disgusting and loathsome, as to deserve the condemnation and unqualified contempt of every member of the profession; and should receive his notice only, in that he should endeavor to pull off the mask from their hideousness and show them up as the offspring of rascality and breeders of folly, disease and death. They are to be condemned because they are manufactured to *sell* and not to cure, and consequently no reliance can be placed in them. Because, also, many of them, are filthy, immoral and murderous. But the reason which should most influence *us* in their condemnation, is, that we know not what they contain.

We learn from pathology that all diseases are not the same, neither does the same disease affect all systems in the same way. The same disease does not manifest itself alike in a lymphatic and a sanguine-nervous temperament; in a robust body, and a frail, fragile frame; in one who labors hard in the open air, and one who sits and sews in a close, dark room. An old physician long ago, found that what cured the Dutch blacksmith killed the French tailor, and it is upon this principle of applying proper remedies to the system in question, that we should expose the absurdity, and condemn the use of the wholesale patent death which is bottled up and sold as "safe and certain cures," under the pleasing, unique soubriquet of patent medicines.

But there is a host of isms and pathies which we should no less repudiate. Scientific medicine is opposed by every possible scheme which it is in the power of the quack's brain, I'd like to have said crack-brain, to invent. We have the systems called Eclectic and Botanic, Isopathy and Hydropathy, Urinopathy and Homœopathy, *et id omne genus*, all setting themselves up against medical science.

Those which are the most prominent among these pathies are steamopathy and homœopathy. Of the former, all I need say, is, that its advocates are fast leaving off the old name and old style of practice, and are giving *concentrated medicines*, and practicing the *reform practice.* And this reform amounts about to the advice Abernethy was once said to have given a lady, to cure her bad smelling breath. Of homœopathy, which is quite a rage just now, it will be well, perhaps, to speak more at length, for, of all the humbug quackery, from the cutting off the finger nails every Friday, to keep off the toothache, up, I consider this the climax.

Hahnemann's magnificent (?) system was founded on two theories—the first was, "similia similibus curantur," or "like cures like." The other was "the value of a medicine was enhanced by division and subdivision, trituration and retrituration," With regard to the first theory I affirm, such a principle has never yet been proved to be a law of therapeutics, much less the foundation law of all practice.

The facts on which homœopaths rely most, are better explained differently. Behold one example—warm water is dangerous in thawing a frozen nose, while it may be preserved by cold water and snow. What boy does not know how to explain this? what negro but knows to warm his benumbed limbs *gradually* and not suddenly?

In such quibbles do they repose their great, their only maxim, "similia similibus curantur," which being interpreted, signifies, if a man is puking himself to death, give him an emetic; if he falls down a flight of stairs and breaks his leg, kick him down another flight, and he'll get well. The doctrine of infinitesimals is another *foundation principle* of the Dutchman's humbug. This dogma has been beautifully illustrated by a late writer, thus: "If a drop of cologne be put into a hundred drops of water, and shaken a hundred times it will have a hundred times more powerful effect than it had at first;" and that this process may be continued till it becomes so strong that it would impregnate Lake Superior, and if one drop of this be taken it will result in most terrible effects on the human frame, which will go on developing themselves for a month. Why, after taking this infinitesimal, a man will actually scratch his head—if there are inhabitants in his hair—go to sleep after dinner, kiss his baby, or be guilty of some equally heinous offence, and all the result of taking the ten millionth part of a drop of cologne.

It is gravely asserted by Teste, in his Materia Medica of Homœopathy, that the decillionth part of a grain of a white pebble will produce such symptoms as, ill humor—sort of intoxication in the evening—vertigo, obliging one to hold on to something—headache from the nape of the neck to the vertex, hindering sleep—sweat about the head in the evening—agglutination of the lids in the morning—shocks in the teeth, when sucking at them with tongue—pain at the pit of the stomach, when pressing on it—tendency to take cold, when uncovering the feet—voluptuous and furious itching, when scratching a little spot on the sole of the foot—vascular excitement and thirst, after drinking a little wine—a good many dreams every night—bellyache, with diarrhœa—wetting the bed at night—itching of the vulva, etc., etc., etc.

This from me sounds like a caricature designed to cast a slur on Hahnemanism, but it is not so; these *symptoms* are veritable quotations from a standard Homœopathic Materia Medica, and they are only a few taken from three or four pages of the same sort. Can such frivolous nonsense that a decillionth of a grain of sand, common salt or oyster shell should produce such manifest effects be seriously believed? Verily, when we read such things we are constrained to say with Shakspeare, "judgement thou art fled to brutish beasts and man has lost his reason."

Such are the great *theories* which are to overthrow all the facts which medical science has established. Doctor Edward Warren, of N. C., in his Essay on Influence of Pregnancy on the Development of Tubercles, which I have seen since writing this, has made some admirable remarks which I must introduce, on these boasted dogmas of Hahnemann. He says:

"These false doctrines have already reached the climax of their glory; the world begins to realize that it has been deluded long enough by the maxims of this mistaken philosophy; the period for discussion has gone by, and it is only necessary to make a plain statement of the whole matter, and then leave it to the common sense of mankind, in order to secure the complete overthrow of this pernicious system.

At Leipsic, which has been the headquarters of Homœopathy, the only hospital devoted to that system, contains but six beds, and all of these are not usually occupied.—In Paris M. Andral put it to the test of experience in one of the general hospitals, and the result was a total failure. He treated one hundred and forty patients in the presence of the homœopathists themselves, adopting every requisite care and precaution, and, yet, in not one instance was he successful. In Russia the Grand Duke Michael invested a German homœopathist with full powers to test its merits, and in two months the experiment was pronounced unsatisfactory by the government, and discontinued. In Naples a trial was made by the royal order, by which it was established, not only that Homœpathic treatment produced no effect on disease, but that it was positively injurious, for the reason that it prevented the employment of remedies by which the patients might have been cured. In London there are at present but two homœpathic hospitals, one of which is about closing for want of funds, and the other is in a declining state. Thus has the system of Hahnemann proved a failure when tested practically, and is now everywhere on the decline. Theoretically, it has not been more successful, as must be admitted by every unprejudiced mind. The Homœpathists have failed to demonstrate either that medicinal powers do produce an artificial malady, similar to the natural affection; that the organism only remains under the influence of the medicinal disease, that the artificial disease is of short duration, or that all the effects can only be produced by selecting an agent which produces results similar to the symptoms, and hence, their doctrines have not only been impugned by Ran, Shrœn and Griesselich, but repudiated as illogical and visionary by the most intelligent observers throughout the world."

In view of the developments here made, we must say, that though there may be some good and some truth (a little to be sure) in most of the other quack systems, yet, in this impudent farrago of trash, there is not even a Homœopathic quantity of either good or truth.

There is absolutely nothing but falsehood and impudence in the system. Both its positive and negative phases are bad, and bad continually, and it is an insult to an educated mind to offer for its consideration such foolhardy absurdities.

The next aspect we take is a view of what we may call *M. D. quackery.* The M. D. quackery manifests itself, of course, in the regular profession, and it will be well for us to know it when we see it. First, then, as a specimen of this species, we notice the fact that many a *fellow* who has by some means made his way through a Medical College is puffed up with the idea that at the talismanic word diploma all the ills that came out of Pandora's box are to be driven to the nethermost parts of the earth, and all the people shall fall down and worship him who bears the precious sheep skin. You always know this fellow by his bluster about his diploma, and his utter disregard of qualification, if he is only satisfied you have a diploma and say you are a regular. The fact is, diploma has haunted his otherwise vacant cranium, till he thinks the whole world ought to be enveloped in sheep skin, and *his* name should be written on it. Such men are quacks—they have but one idea. Another of the same nature is the routine practitioner—he who gives the same remedy for every disease.

Again, we have a specimen in those who desire to wrap the profession of medicine in mystery, considering its beauties too sacred for the common gaze ; and, while they are thus busy in keeping others out of the charmed inclosure, like the door-keeper of a circus, they see very little of what is going on inside.

Another quack specimen is found in some of our Colleges. The man who would sell a diploma to, or confer the degree of M.D. on, a man who has *no* claim to such an honor, is a maudling, dishonest Quack, coming before us under the fair colors of Science.

Another, and perhaps the last specimen of quackery, we shall notice, is the M. D. who is always running into extremes ; such, for instance, as calling himself an Allopath, or Alleopath, because, forsooth, he is opposed to Homœpathy. In the name of reason, does it follow because a man denies the dogma *similia similibus curantur*, he must adopt the equally silly *contraria contrariis curantur*. Such is the charge Homœpathists have had the effrontery to make upon scientific physicians ; and, alas ! many an M. D. has been weak and childish enough to acknowledge to it, and style himself an Allopath. There is just as much reason in a man's calling himself a Dirt-o-path—one who cures by administering dirt,—because he is opposed to hydropathy, or water-cure; as there is in calling one's self an Allopath because he is opposed to Homœopathy. Does not every physician know that Medical Science is not a pathy, any more than are all the pathies Medical Science. I earnestly hope that the day is not far distant when medical men will cease to reject cold water, or hot water, as a remedial agent, *because* some extremist uses it. When they will cease to condemn any remedy, or any principle of a pathy, *because* they are the bantlings of a pathy. But, will learn to reject and condemn any and every remedy, so called, on principle; because it is unscientific, and cannot be established by reason, and be able, at all times, to give the reason why he does condemn them. By making rigid Science, and not prejudice, the standard which governs the medical mind, M. D. quackery will soon be rooted out from among us, and all our energies can then be given to the demolishing of humbug quackery.

But I must, for a moment, consider the causes of success of this humbug quackery. The main causes of its rapid advance and stronghold upon the popular mind are: Its advertisements, certificates, and puffs are so widely disseminated through the country by a subsidized news paper press. It has become a business, and its artful practitioners have interwoven it with other businesses, so as to affect the pocket, and, of course, the influence of all through whom they operate. Nostrums are often certified by M. D.'s, by lawyers, and demagogues, and even gentlemen of the white cravat forget their ministerial dignity, and testify to the efficacy of a filthy compound, whose very mention would cause a blush upon the face of their wives or daughters. And another is the facility with which the common people mistake a sequence for an effect. They see their neighbors, take these stuffs, and get well, consequently, they reason, the medicine cured them; but, unfortunately, if they die from taking them, they never attribute the result to their villainous nostrums, but they think their time had come. Such are some of the prominent causes of the success of humbug-quackery.

And now I will conclude by glancing at the proper means by which quackery may be killed off. We need not expect to do it by calling the people fools, or by simply ridiculing it. Nor can we legislate it out of existence. The only possible way by which we can accomplish this desirable end is to enlighten the masses in the great truths of physiological and pathological Science; by disseminating information on the wonders of the human system and the laws of health, and the phenomena and facts of life, as they manifest themselves.

I have shown that these nostrums succeed mainly thro' the influence of the newspaper press. They are puffed, and blowed, and lauded, and no one contradicts them before the people. Now, I believe that the only available medium by which we can accomplish this general education is the press,—through a newspaper disseminated through the land. There is no hope of reaching the diseased point through the periodical monthly medical publications. It must be done by the same kind of a paper—a family paper—that the mischief is done by. The family is the only medical paper which will be patronized by the masses; hence, there should be such a publication, to accomplish this purpose. True, something may be done by private effort and private explanation, but I am convinced, as I have said, that the press is the lever by which we are to move this "mountain of sin." But I am not alone in this view of enlightening the people. I have the concurrence of the learned Professor of Chemistry in Jefferson Medical College. In his late valedictory address before the Graduating Class, delivered March 15th, 1859, Professor Bache says: "I need not caution you against those other quacks, who make no pretensions to Medical Science, but sell their nostrums with an unblushing effrontery to all who are willing to buy them. It is ignorance on the part of the community that makes the quacks, not the quacks the ignorance by which they live. Hence, to eradicate quackery, physicians should enlighten the masses as to the more obvious principles of physiology and pathology, or the laws of the human organization in health and disease. By taking this course, the demand for quacks would be lessened or destroyed, and, consequently, the supply. Unfortunately, the profession has stood too much on its dignity. It has too frequently made a mystery of medical science, and the appliances of our art. If we would oftener condescend to make explanations to our patients or their friends; if we would encourage the teaching of the elements of physiology in our schools, and of kindred subjects, by popular lectures, to adults, we might hope to dispel some of the ignorance which forms the sole sustenance of quackery. It is, indeed, true that the ignorance of the present day is not quite so great as it was centuries ago; yet the ignorant of modern times are much more readily reached by the forgeries and false statements of empirics, disseminated over the land by the subsidized newspaper press. In times past the weapon that inflicted a wound was dressed with a healing ointment, as well as the wound itself; and the rusty nail that pierced the foot was greased and hung up the chimney, to ward off an attack of lockjaw. At present, the royal touch is no longer believed in as a cure for scrofula, and the natural bone-setter is almost an extinct animal. Still, quackery is rife to an alarming degree, and is patronized by the rich as well as the poor; and in our day its mischiefs are far more diffused than they were in the olden time, owing to the specious disguises it puts on, and by reason of the activity of the press. The cause of this enormous evil, we repeat, is ignorance in the masses, whether they live in fine houses or in hovels, and the remedy is to enlighten them."

When this has been done, quackery shall be buried beneath the heaped-up contempt of the world, and the firm, everlasting establishment of the temple of Scientific Medicine shall be heralded through the universe in a pæan of a thousand jubilant notes.

